# Influence of Topographic Factors on the Characteristics of Gully Systems in Mountainous Areas of Ningnan Dry-Hot Valley, SW China

**DOI:** 10.3390/ijerph19148784

**Published:** 2022-07-19

**Authors:** Yuxin Cen, Bin Zhang, Jun Luo, Qingchun Deng, Hui Liu, Lei Wang

**Affiliations:** 1 School of Geographical Sciences, China West Normal University, Nanchong 637009, China; cenyuxin9821@163.com (Y.C.); envgeo@163.com (B.Z.); luojunxmx@126.com (J.L.); dqccwnu@163.com (Q.D.); huatanhe@cwnu.edu.cn (H.L.); 2 Sichuan Provincial Engineering Laboratory of Monitoring and Control for Soil Erosion in Dry Valleys, China West Normal University, Nanchong 637009, China; 3 Liangshan Soil Erosion and Ecological Restoration in Dry Valleys Observation and Research Station, China West Normal University, Nanchong 637009, China; 4 State Key Laboratory of Resources and Environmental Information System, Institute of Geographic Sciences and Natural Resources Research, University of Chinese Academy of Sciences, Beijing 100048, China

**Keywords:** gully erosion, topography conditions, spatial pattern, hierarchical clustering method

## Abstract

A gully system is an important indicator that reflects the development of regional topography and landforms, and topography is one of the most important factors affecting the development of gullies. However, at present, research on the impact of topography on the development of gully systems in the mountainous area of Ningnan dry-hot valley still needs to be strengthened. In order to study the characteristics of gullies and the influence of topography on the development of gully systems, based on both the visual interpretation of remote sensing images and field investigations, five topographic factors (elevation, slope gradient, aspect, relief, and dissection) were employed and three gully erosion indexes (gully length, density, and frequency) were calculated. The geographical information system was used in this study to carry out the spatial analysis, Ward’s hierarchical clustering and correlation analysis. Results showed that the development of gully systems is greatly affected by the degree of relief and dissection, and there is a significant positive correlation (*p* < 0.01; *p* < 0.05), while elevation, slope gradient and aspect have little influence on it. Analysis of the gully systems showed that the gully erosion is the most intense in the area with an elevation of 2800–3200 m and slope gradients ≥ 38°. Furthermore, the degree of erosion on shady slopes was greater than that on sunny slopes. These results will help us to understand the spatial distribution and formation of gully systems in mountainous areas.

## 1. Introduction

Soil erosion is a destructive environmental problem that affects social and economic development, agricultural production and sustainable use of natural resources [1,2]. Gully erosion is one of the most serious types of soil erosion, especially in mountainous areas, which inputs a large amount of sediment into regional rivers every year [3,4]. Gully erosion is the process of overland flow converging into what are initially, interconnected micro-depressions that form a natural drainageway [5]. The understanding of gully erosion damage is reflected in the calculation of the gully erosion amount and erosion rate [6,7]. At present, studies on gully erosion have been expanded from gully development and erosion mechanism [8,9], influencing factors and prevention measures to the studies on gully methods and models [10,11,12], and the effects of the ecological environment [13]. Studies of gully erosion mostly use the gully length, width, depth [14,15], density, and erosion modulus to describe the severity of gully development [16]. Research on gully systems has mainly focused on the direction of the gully, the valley profile [17], the fractal characteristics of the gully head [18] and the spatial distribution of gullies [19].

Gully erosion is the result of a series of interrelated processes that depend on the environment [20]. The factors related to gully erosion can be divided into five categories: topography, climate, land management, soils and hydraulics [21,22]. Topographic factors were widely focused on because the erosive effects of water flow are related to the topography [23,24]. The combined influence of hydrology and topography affects soil stability by reducing the shear strength and soil anti-erodibility [25]. The influence of topography on the development of gullies is reflected in the slope length, slope gradient and catchment area [26]. Slope length provides sufficient runoff for the development of the gullies, indirectly enhancing the erosive power of water. Slope is the key factor affecting runoff shear stress, and it is also the key topographic factor of gully formation [5]. The characteristics of gullies vary with the topographic position and slope gradient [27]. The critical conditions and spatial pattern of gully erosion have been determined by studying the correlation between the catchment area and other topographic factors [28,29]. In addition, the varied land use and management practices in different countries over different time periods have led to different effects, so human influences are often one of the driving forces in the evolution of gully [30].

The description of topographic features based on digital elevation models (DEMs) is the focus of current topographic research [31,32]. To study the development of gullies and to establish gully erosion models, topographic maps and large-scale satellite images (Landsat, Modis, Terrain data of SRTM, etc.) were usually used as data sources of remote sensing and geographical information system technologies [19]. Based on these data and combined with field surveys [33], the topographic factors (e.g., altitude, slope gradient, aspect, relief and dissection) and spatial distribution of gullies can be analyzed effectively, the relationships between topography and the distribution of gullies can be established [34], and the formation and development of gullies can be revealed. At present, the quantitative relationship between gully distribution and environmental factors has been widely studied. For example, the spatial distribution of gullies on a large regional scale, the correlations between gullies and geological conditions [35], the impact of land use on the development of gullies [36,37], the development and distribution of roadside gullies, the development of gullies under different watershed conditions [38], and the impact of gullies on eco-environmental quality [39]. These works were of great significance for the evaluation of gully erosion control [40].

An exploration of the effects of topography on the development of gully will help us to understand the characteristics and formation of gullies, and provide a scientific basis for the evaluation, prevention and control of the hazards caused by gullies in mountainous areas. At present, the research on the relationship between topography and gully erosion mainly focused on the relationship between a single topographic factor and gully erosion or compared the contribution of multiple topographic factors to the development of gully erosion [41]. Quantitative analysis of gully development in different geomorphic units and the influence of topographic factors on gully system development were rarely reported. The superposition effect of different factors on gully development and the effect of different geomorphic units on gully evolution need to be further studied. Therefore, the research objectives of this study were to (1) quantify the intensity of gully erosion under different topographic factors, (2) reveal the relationships between topographic factors and gully development, and (3) find out the topographic conditions with the most serious gully erosion.

## 2. Study Area

**Figure 1:**
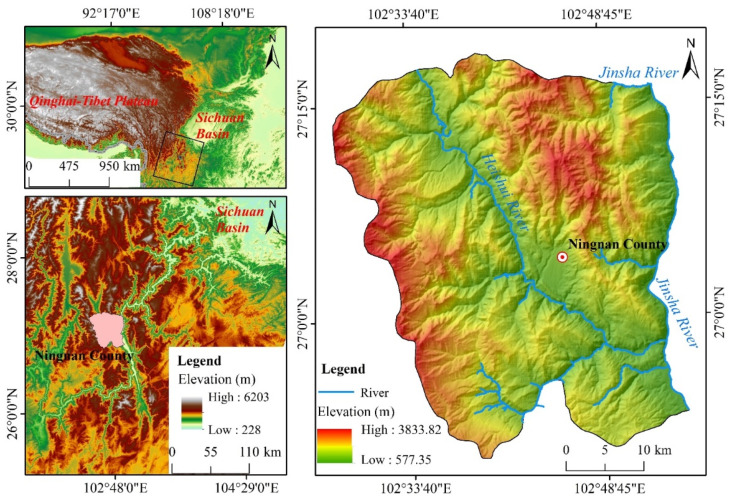
Location and elevation maps of the study area. Ningnan County is located on the southwestern edge of the Sichuan Basin in China, and its northwest region is the Qinghai–Tibet Plateau.

Ningnan dry-hot valley is located on the southwestern edge of China’s Sichuan Basin, on the western side of the mid-reaches of the Jinsha River (Figure 1). It developed in the mid-section of the Sichuan–Yunnan meridional structural belt [42], at the junction of the Ninghui fault zone and the north–south fault, and is low in the south and high in the north. The geographical location is (102°27′–102°55′ E, 26°50′–27°18′ N) and the total area of the study region is 1685.95 km². The area has a subtropical monsoon climate and is affected by the westerly circulation, the southwest monsoon and the western Pacific warm and humid monsoon, leading to a dry–hot climate; the forest coverage rate is 23.7% [43]. The fragility of the ecological environment caused by the climatic characteristics, coupled with the effects of human activities, has broken the surface of the Ningnan Mountain area and led to the active development of gully erosion. The average density of gullies is now 2.82 km/km^2^, and gully erosion is an important method of soil erosion in this region.

## 3. Materials and Methods

### 3.1. Topographic Factor Extraction

The 30 m-resolution original elevation data of the study area were obtained from the Shuttle Radar Topography Mission (SRTM) DEM, and the coordinate system is GCS_WGS_1984. Based on the DEM data for this area, the elevation, slope gradient, aspect, relief and dissection factors affecting the development of gullies using the ArcGIS (version 10.2) spatial analysis function were extracted (Table 1).

### 3.2. Gully System Parameters

The gullies were derived from the visual interpretation of remote sensing images (images from Google Earth: © 2022 Google, Digital Globe). The use of computer interpretation alone would have reduced the accuracy of the interpretation as a result of the presence of both vertical and horizontal gullies and the complex topography. Google Earth high-resolution remote sensing images of the study area were vectorized through visual interpretation and the gullies extracted. Field verification was then carried out at the relevant location and combined with visual interpretation to give the spatial distribution of the gullies.

The specific steps for the visual interpretation of remote sensing images were as follows: (1) import the downloaded remote sensing image maps into ArcGIS 10.2 for gully vectorization; (2) create a shapefile working layer in ArcGIS; (3) select lines for all types of gully element; (4) set the spatial reference coordinate system consistent with the remote sensing images (GCS_WGS_1984). Artificial visual interpretation was carried out based on the image, reference materials and field observations and the gullies in the research area were vectorized according to the principle of the main gully first, then the branch gully, and according to the direction from the gully head to the gully outlet. Topological inspection of the visual interpretation results, geometric calculation of the gullies and calculation of the length of each gully were used as the basic attribute data.

The indexes of gully length, density, and frequency were established to analyze the development of gullies and describe their spatial differentiation characteristics based on the interpreted vector data of gullies in the Ningnan dry-hot valley. The relevant data from ArcGIS was extracted and the indicators needed to measure the development of the gully systems were calculated. The indicators to be extracted and their definitions and calculation processes were as follows.

The gully length was defined as the total length of the gullies contained in a certain study area. The gully density was defined as:

(1) $$d=\sum_{j=i}^{m}L_{j}/S_{0}$$

where *d* was the gully density (km/km^2^)—that is, the total length of the gully per unit area in a certain study area. *L* was the total length of the gully in a certain area (km) and *S*_0_ was the total area of a certain study area (km^2^).

The gully frequency was defined as:

(2) $$f=\sum_{j=i}^{n}N_{j}/S_{0}$$

where *f* was the gully frequency value—that is, the ratio of the total number of gullies in a certain study area to the surface area—and *N* was the total number of gullies in a certain study area.

The accurate change trend of the gullies with the topographic factors by analyzing the DEM topographic data of the study area was obtained and then digitizing it. Superimposing these data with the interpreted gully vector data, forming the space of each topographic factor in the gully erosion process distributed database. Then, the spatial differentiation characteristics of gullies of different elevations, slope gradients, aspects, reliefs and dissections were analyzed.

### 3.3. Ward’s Hierarchical Clustering Method

Ward’s method in SPSS 21.0 (IBM Corp., Armonk, NY, USA) was used with the gully parameters as analysis indicators to re-quantitatively determine the close relationship between the samples of topographical factors. First, considering each of the *n* samples as one class and stipulated that the distance between the sample and the sample was equal to the distance between the class and the class. The sum of the squared deviations of the samples of the same class should be small and the sum of the squared deviations of the class and class should be larger. The distance between samples must use a Euclidean distance. Then, the two classes with the smallest distance were chosen and combined into a new class. The distance between the new class and other classes was recalculated and then the distance between the two closest classes was calculated. These were combined until all the samples were classified into one category to obtain the rescaled distance cluster combination of four topographic factors.

Then, *n* samples were divided into *k* categories (G_1_, G_2_, …, G_k_) and *x_it_* was used to represent the variable index value vector of the *i*th sample in G_t_. If *n_t_* represented the number of samples in class G_t_ and *x_t_* represented the center of gravity of G_t_, then the equation for the sum of the squared deviations of the sample in G_t_ was [44]:

(3) $$S_{t}=\sum_{i=1}^{n_{t}}(x_{it}-\bar{x_{t}})\prime (x_{it}-\bar{x_{t}})$$

The equations for the sum of squared deviations within all classes were:

(4) $$S=\sum_{t=1}^{k}S_{t}=\sum_{t=1}^{k}\sum_{i=1}^{n}\left(x_{it}-\bar{x_{t}})\prime (x_{it}-\bar{x_{t}}\right)$$

## 4. Results

### 4.1. Reclassification of Topographic Factors

**Figure 2:**
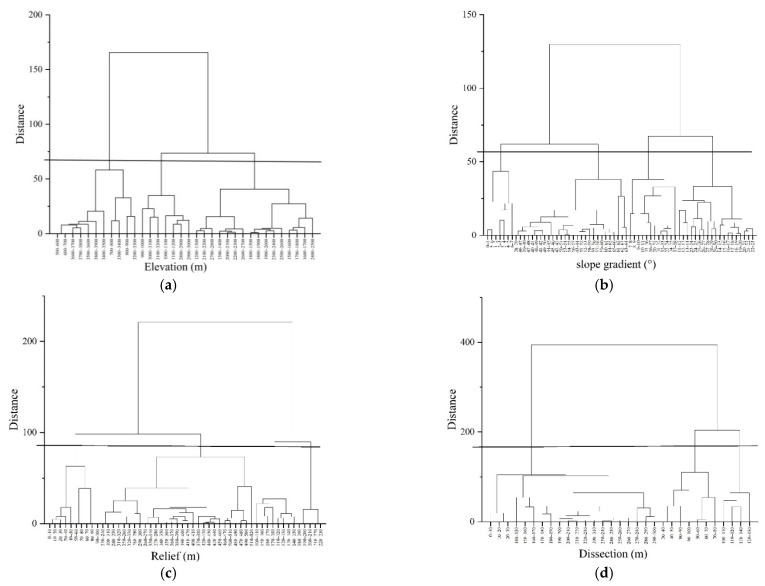
Tree diagram of hierarchical clustering in topographic factors. The horizontal line in the figure is the distance value for classification. The closer the distance, the more similar the gully erosion forms in the topographic range classification. (**a**) Tree diagram of hierarchical clustering in elevation; (**b**) Tree diagram of hierarchical clustering in slope gradient; (**c**) Tree diagram of hierarchical clustering in relief; (**d**) Tree diagram of hierarchical clustering in dissection.

The topographic factors were reclassified by Ward’s hierarchical clustering method with gully parameters as analysis indicators (Figure 2), then the continuous topographic factors were divided in the same category. The elevation and slope gradient were reclassified into five categories and the relief and dissection were reclassified into four categories (Table 2). The aspect is still classified as in Table 1.

### 4.2. Characteristics of Gully Systems

**Figure 3:**
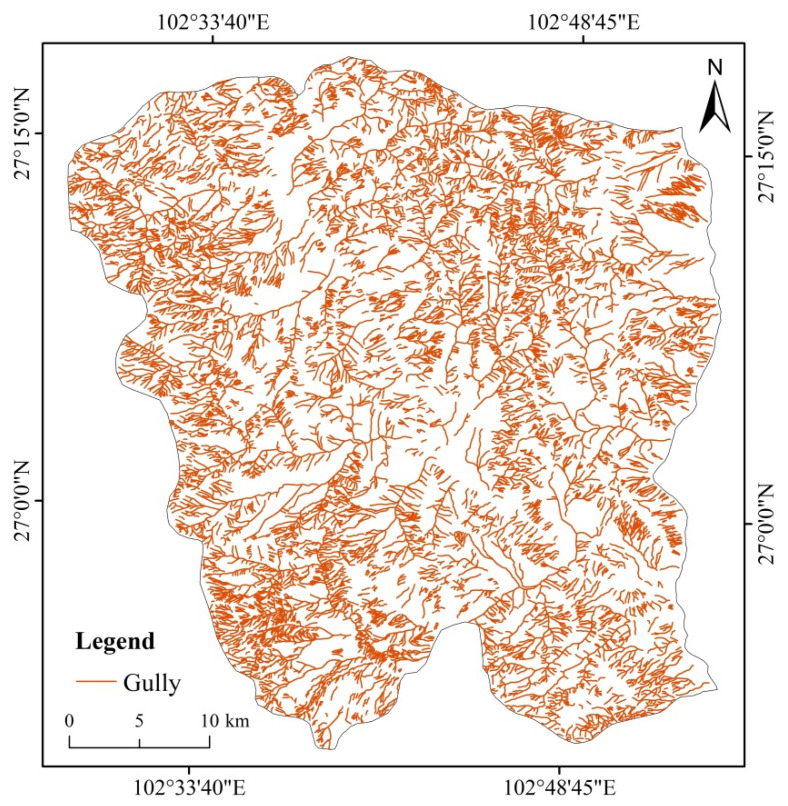
The distribution characteristics of gullies in the study area by visual interpretation of remote sensing images.

The current situation and characteristics of gully development based on the visual interpretation attribute data of gullies in the Ningnan dry-hot valley were analyzed. The gullies were widely distributed in the Ningnan mountainous area and the development of gully erosion was uneven. The distribution of gullies was more concentrated in the western and northern regions than in the central and eastern regions. Although there were also gullies in the lower lying areas in the center, the number was relatively small (Figure 3).

### 4.3. Variation of Gully Parameters with Reclassified Topographic Factors

#### 4.3.1. Elevation

**Figure 4:**
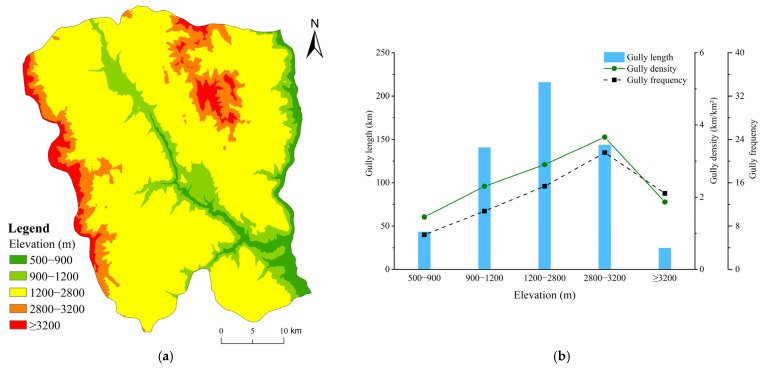
(**a**) Distribution of reclassified elevation ranges; (**b**) Characteristics of gully system parameters (gully length, density, and frequency) under different elevation ranges.

The elevation of the study area increased with the middle valley area to the eastern and western mountains. The elevation ranged between 577.35 and 3833.82 m, and the average elevation was 1946.28 m. The difference in elevation was large and the study area was hilly and mountainous. The statistical results for the distribution of gullies were obtained and the development characteristics of different elevation categories based on the reclassified elevation range of Ningnan dry-hot valley (Table 2). The intensity of gully erosion first increased and then decreased with the increase in elevation (Figure 4). The most intense gullies were developed in the 2800–3200 m elevation range, with a gully density of 3.66 km/km², in which gullies develop frequently and with a gully frequency of 21.56. The average gully length in the elevation range 1200–2800 m was 215.77 km, and the number of gullies in this elevation range was the largest, with the longest total gully length, but this elevation range is the widest. The corresponding average gully density in this elevation range was 2.90 km/km², with an average gully frequency of 15.36. The elevation ranges 500–900 m, ≥3200 m and the 900–1200 m elevation range were very low cluster erosion area with weakly developed, gully density was 1.45, 1.87, 2.30 km/km², respectively. Overall, an increase in elevation promoted the development of gully erosion in Ningnan dry-hot valley.

#### 4.3.2. Slope Gradient

**Figure 5:**
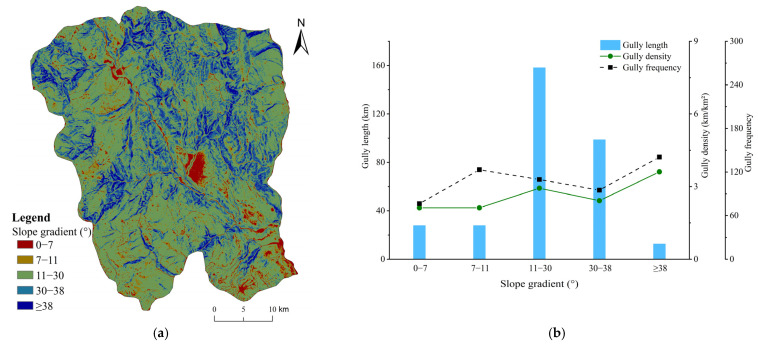
(**a**) Distribution of reclassified slope gradient ranges; (**b**) Characteristics of gully system parameters (gully length, density, and frequency) under different slope gradient ranges.

The range of slope gradients in Ningnan dry-hot valley was 0–72°, with an average slope gradient of 23.84°. The distribution of gullies from slight to steep slopes was very different. The distribution and development characteristics of gullies of different slope gradient categories based on the reclassification range of slope gradients in Ningnan dry-hot valley (Table 2) were obtained. Slopes gradient of ≥38° had the most intensely developed area of gullies in Ningnan dry-hot valley (Figure 5). The average gully density was 3.61 km/km², and the gully frequency was 140.71. Additionally, gullies were strongly developed on slope gradients of 11–30°, and the average gully density was only 2.93 km/km² and the gully frequency was 109.71. Stable gully erosion developed in the 0–11°, and 30–38° slope gradient zone, and the average density was 2.12 and 2.42 km/km². The total gully length changed drastically in each slope zone.

#### 4.3.3. Aspect

**Figure 6:**
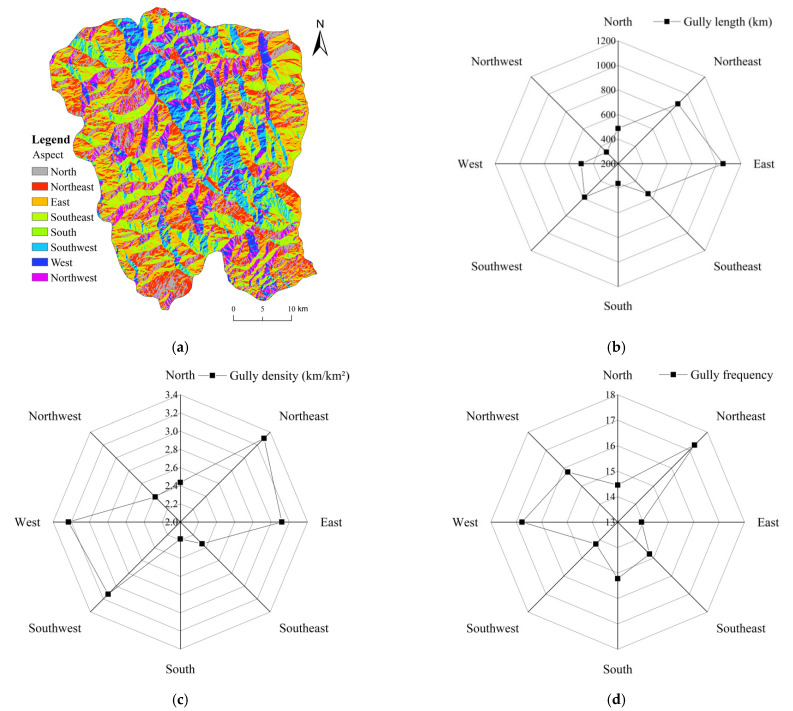
(**a**) Distribution of aspects; Characteristics of gully system parameters (gully length, density, and frequency) under different aspects: (**b**) Gully length; (**c**) Gully density; (**d**) Gully frequency. It reflects the distribution of gully parameters in different aspects.

Figure 6 shows the coupling relationship between the development characteristics of gullies and slope aspect. The gullies have different distribution characteristics on different aspects (Figure 6). The spatial variation trend of gully length on the western, southwestern and southern slopes was relatively gentle, whereas the gullies had a larger distribution on the northeastern, eastern and southeastern slopes. Gullies were strongly developed on the western, southwestern, eastern and northeastern slopes. The gully density was highest on the northeastern slopes, reaching 3.30 km/km². The gully density was smaller on the northwestern, northern and southeastern slopes. The southern slopes had the lowest gully density. Based on the statistics, the total gully lengths of sunny, semi-sunny, semi-shady and shady slopes were 945.70, 1045.02, 1386.59 and 1374.48 km, respectively, and the average gully densities were 2.68, 2.69, 2.93 and 2.90 km/km², respectively. The distribution characteristics of gully erosion intensity were shady slope > sunny slope and semi-shady slope > semi-sunny slope. The density of gullies in the shady slope area was 2.92 km/km² and the gully density in the sunny slope area was 2.69 km/km². These statistical results show that the degree of gully erosion on shady slopes was greater than that on sunny slopes and that the gully erosion intensity increased on shady slopes.

#### 4.3.4. Relief

**Figure 7:**
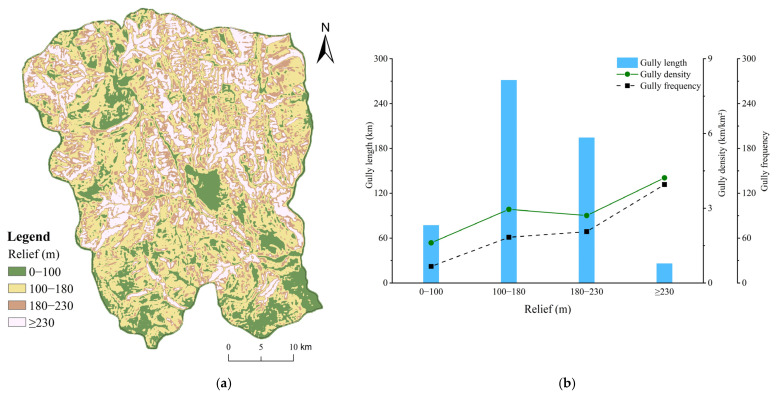
(**a**) Distribution of reclassified relief ranges; (**b**) Characteristics of gully system parameters (gully length, density, and frequency) under different relief ranges.

The range of relief in Ningnan dry-hot valley was 0–580.80 m with an average of 164.30 m. The gullies were concentrated in the mountainous areas. The distribution and development characteristics of gullies of different relief categories based on the reclassification range of relief of Ningnan dry-hot valley (Table 2) were obtained. The overall trend of gully density and gully frequency in each relief zone increased monotonically (Figure 7). Gullies become more frequent and more densely distributed with the transition from the plains to the mountains. The 0–100 m relief zone had weak gully development, with a gully density of 1.61 km/km²and gully frequency of 22.13. Gully development was relatively stable in the 100–230 m relief zone, this relief zone had a large area with a large number of gullies. The relief zone ≥ 230 m was the most eroded belt in Ningnan dry-hot valley and average gully density was 4.22 km/km² and the average gully frequency was 131.71. The larger the relief in the study area, the more frequent the gullies, the denser the distribution and the more severe the erosion.

#### 4.3.5. Dissection

**Figure 8:**
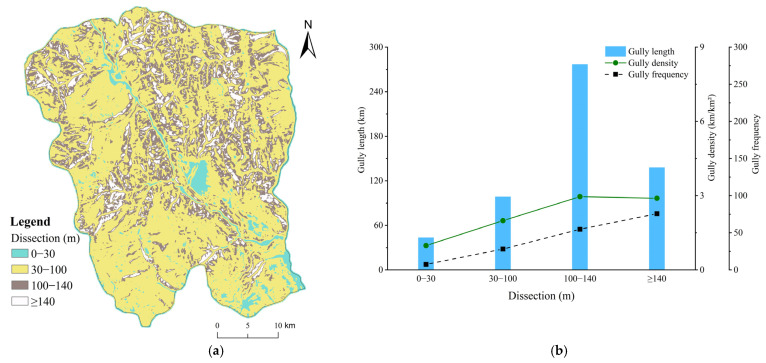
(**a**) Distribution of reclassified dissection ranges; (**b**) Characteristics of gully system parameters (gully length, density, and frequency) under different dissection ranges.

The degree of dissection in Ningnan dry-hot valley ranged from 0 to 340.77 m, with an average degree of dissection of 81.82 m. The distribution and development characteristics of gullies of different dissection categories based on the reclassification of dissection of Ningnan dry-hot valley (Table 2) were obtained. Gullies developed most strongly at 100–140 m and ≥140 m, the average gully density were 2.96 and 2.90 km/km². In these two areas, gully erosion develops most violently, and the average gully frequency was 54.78, 75.81, respectively (Figure 8). Most gullies were distributed in the 100–140 m dissection zone and the weakest area of gully development was at 0–30 m. The gully density was high in the topographic belt with a large degree of dissection. This indicates that the gully density was higher in the area with a greater surface fragmentation and that gully erosion led to more serious soil erosion. The pattern of gully erosion was relatively uniform where the surface was severely cut; unless there were large natural or anthropogenic interference factors, the gully erosion pattern was generally stable.

### 4.4. Correlations between Topographic Factors and Gully Parameters

Spearman correlation analysis was performed on the extracted topographic factors and gully parameters (Table 3). Elevation was significantly positively correlated with gully frequency (*p* < 0.01), but had no significant correlation with gully length and gully density. This means that the higher the elevation, the denser the gully development. The slope gradient had a significant negative correlation with gully length (*p* < 0.01), a significant positive correlation with gully frequency (*p* < 0.05) and no obvious correlation with gully density. As the slope gradient increased, the gullies become more frequent, and the area occupied by the slope gradient decreased. The gully length and slope gradient were therefore negatively correlated. The aspect and gully length, density and frequency all failed the significance test, indicating that the aspect has little effect on gully erosion, and there was no obvious correlation between them. Both relief and dissection were significantly negatively correlated with gully length (*p* < 0.01) and had a significant positive correlation with gully frequency (*p* < 0.01). There was a significant positive correlation with gully density (relief *p* < 0.01, dissection *p* < 0.05), indicating that gully development became more intense with an increase in relief and degree of dissection because the proportion of the area occupied by each step of the topographic factor decreased. The gully length decreased, whereas the gully density and the overall gully erosion increased.

## 5. Discussion

### 5.1. Influence of Topographic Factors on Gully Parameters

The results of the correlation test between the topographic factors and gully parameters showed that variations in relief and dissection had a severe impact on the development of gullies and there was a significant positive correlation. By contrast, elevation, slope gradient and aspect had relatively little influence on the development of gully erosion in this area. An increase in relief and dissection was accompanied by an increase in soils and rocks leaving their original locations under the action of gravity, and rainfall added hydraulic action and increased soil erosion. This is consistent with previous studies that height difference has a greater impact on gully development than altitude [45].

**Figure 9:**
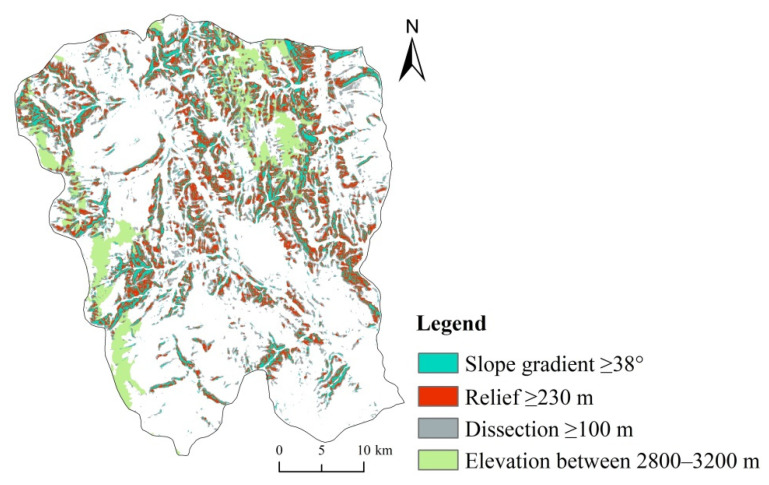
Area with intense gully erosion under various topographic factors. Most of the areas covered by the four indicators overlapped, indicating that the development of the gully was related to the topography.

The intensity of gully erosion first increased and then decreased with increasing elevation in this study, which is similar to the previously research results of typical rolling hill Mollisol region of Northeast China, where gully erosion decreases near the top of the slope [46]. Study found that gullies were strongly developed where the slope gradient was larger because the gravity effect was greater on large slope gradients, resulting in the decline of surface soil, and rainfall runoff also caused strong erosion under the effect of gravity. Rapid soil erosion occurred on steep slopes in areas with the greatest gully erosion or in sparsely vegetated wastelands. It is found that the gully erosion areas analyzed by the three topographic parameters of slope gradient, relief and dissection are highly coincident (Figure 9), and they belong to the middle mountain area in terms of landform. Therefore, the landform composed of multiple topographic factors is very important in the development of gully erosion, and follow-up research can focus on the correlation between landform and gully erosion development.

**Figure 10:**
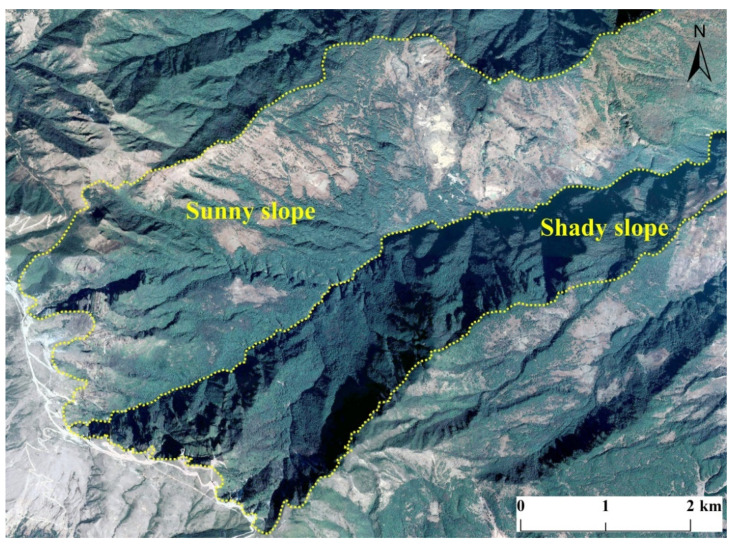
Topographic and vegetation characteristics of typical shady and sunny slopes in the study area, there is a gully between the sunny slope and the shady slope.

The intensity of gully erosion was greater on shady slopes than on sunny slopes and stronger on semi-shady slopes than semi-sunny slopes, and the remote sensing images showed that most of the shady slopes had better vegetation cover than the sunny slopes (Figure 10). Because of the special environmental conditions in the Ningnan dry-hot valley, the sunny slopes had good sunlight, but rapid water evaporation and less vegetation cover, so the gully erosion intensity was expected to be stronger than on shady slopes. However, the opposite results were obtained. This may be related to the combined effects of topographic factors (e.g., relief, dissection, slope gradient, elevation, and other gully erosion factors), which led to a greater formation of gullies on the shady slopes. For example, the gully interpretation results (Figure 10) showed that most of the shady slopes in the study area had steeper slopes than the sunny slopes—that is, the influence of relief and dissection was greater than the influence of slope aspect on gully erosion and even affected the slope. Vegetation cover was better on shady slopes than on sunny slopes, which indicated that the shady slopes in the study area had better moisture conditions. Ningnan dry-hot valley has short rainy seasons, long dry seasons and distinct dry and wet conditions, and surface runoff mostly occurs in summer. However, the long dry season means that the amount of runoff left on the sunny slopes is very limited after infiltration, absorption by vegetation and evaporation, so there is less gully erosion on sunny slopes than on shady slopes. This shows that the original soil moisture content has a greater impact on subsequent erosion [47]. The relationship between aspect and gully erosion therefore requires further research.

### 5.2. Influencing Factors of Gully Development

Ningnan dry-hot valley is a typical mountainous region with hilly landforms. This area is significantly different from the Loess hilly and gully region and plain regions [48,49,50]. Various factors affect the development of gullies were compared and gully erosion are mainly affected by rainfall or surface runoff from rainfall. The emergence of gullies causes hydraulic convergence, which intensifies the hydraulic effect and the development of gully erosion. Gully erosion on the plains and in the Loess hilly and gully region are more affected by rainfall than the mountainous hilly regions. From the perspective of soil and vegetation, the Loess hilly and gully region is bare and the properties of the loess texture are an important cause of soil erosion [51]. In general, the best vegetation cover is found in the mountainous hilly regions, whereas gully erosion in the plains region occurs on fertile soil and is covered by crops [52].

Analysis of the topography showed that the rugged topography was the most important contributing factor to the development of gully erosion in regions with mountainous hilly landforms. Although topographic factors also affect the Loess hilly and gully region and plain region, their effect was reduced. Gully erosion on the plains was mostly a result of the long gentle slopes gathering a large amount of water, which led to the development of a large number of gullies. The plains had fertile soils and many agricultural areas [53], so much of the gully erosion was affected by human activity [54]. The geology, soil, rainfall, hydrology, vegetation and human activities and their interactions all affected gully erosion irrespective of the topography and will therefore be the focus of future studies.

### 5.3. Stable and Active Gullies in Ningnan Dry-Hot Valley

**Figure 11:**
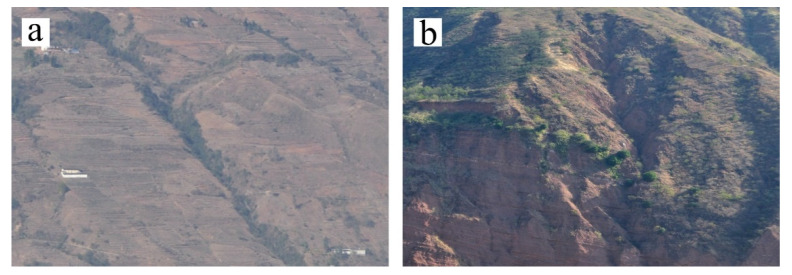
Real view of gullies in Ningnan dry-hot valley: (**a**) Stable gully; (**b**) Active gully.

The existing gullies in Ningnan dry-hot valley can be divided into stable gullies and active gullies (Figure 11). The stable gullies have relatively stable environment through long-term gully erosion and further major changes are difficult, so the gully development is relatively stable [55,56]. In contrast, for active gullies, the gully head are clear and actively developed. In this paper, the two gullies are not distinguished, but they are discussed together. In the process of visual interpretation of remote sensing images and field investigations, high cluster erosion of gullies is mainly distributed in the mountainous areas with few people, whereas the areas with more frequent human activity are mostly areas with low cluster erosion. The results indicate that the topographic conditions are more important than that of human activities for gully development in mountains areas. Topographic conditions have a greater impact on gully erosion in the study area because people mainly live in flat areas, where the development of gully erosion is weak. The active gullies in Ningnan dry-hot valley are often affected more by human activities than the gullies are stable gullies. There are many active gullies in areas with frequent human activities, whereas there are many gullies in the mountainous area are stable gullies. The existing gullies in the mountainous area of Ningnan were mainly belong to the stable gullies.

## 6. Conclusions

There were 6947 gullies in the study area, with a total length of 4804.04 km, and a gully density of 2.82 km/km². The overall distribution of the gullies varied with the gully density gradually increasing from the center to the sides of the basin and from the flat topography of the plains to the mountainous areas. The correlation test for five topographic factors (elevation, slope gradient, aspect, relief, and dissection) and the parameters of the gullies showed that gully erosion was greatly affected by the relief and dissection with a positive correlation, but was relatively less affected by topographic factors such as elevation, slope gradient, and aspect. The study results show that the development of gully has obvious topographic critical conditions, and the landform composed of multiple topographic factors is very important in the development of gully erosion.

## Figures and Tables

**Table 1:** Topographic factor extraction method and classification level.

Topographic Factor	Data Source and Processing Method	Classification Level
Elevation	Based on the 30 m-resolution DEM data of the study area.	The elevation was reclassified based on the actual situation of the study area. The elevation range of Ningnan dry-hot valley is 577.35–3833.82 m, the division range was selected as 500–3900 m and the elevation interval was 100 m.
Slope gradient	Using the DEM data constructed in the study area, the slope tool in the raster surface under the 3D Analyst tool module in ArcGIS 10.2 was used to extract the topographic parameter of the slope gradient in the study area.	The slope gradient range in the study area was 0–72° and the extracted slope gradient data was divided with 1° as the reclassification interval.
Aspect	Using the DEM data constructed in the study area, the aspect tool in the raster surface under the 3D Analyst tool module in ArcGIS 10.2 was used to extract the topographic parameter of the area aspect.	The aspects in the study area were reclassified into nine groups, 0° as true north, increasing clockwise, corresponding to the eight aspects of north, northeast, east, southeast, south, southwest, west, northwest, and flat land (without slope direction) to give a qualitative description of the different aspects.
Relief	Relief = maximum elevation minus minimum elevation (the difference between the highest altitude point and the lowest altitude point in the specified area). Using the ArcGIS platform, the DEM data in the study area with a rectangle as the analysis range was analyzed and the neighborhood radius was gradually increased with an equal interval of 1 m until the relief stopped increasing as they stabilized. The analysis obtained the best statistical unit of relief in this study area and then the field calculator was used to obtain the relief of the study area.	The range of relief that fell into the best statistical unit in the study area was 0–580.80 m and the raster data were reclassified and divided at 10 m intervals.
Dissection	Dissection = mean elevation minus minimum elevation (the difference between the average elevation of the neighborhood of a certain point on the ground and the minimum elevation of the neighborhood reflects the erosion and dissection of the ground). Using the ArcGIS platform, the DEM data in the study area with a rectangle as the analysis range was analyzed. The neighborhood radius was gradually increased with an equal interval of 1 m until the dissection stopped increasing after it stabilized. The analysis obtained the best statistical unit of dissection in this study area and then the field calculator was used to obtain the dissection of the study area.	The range of the dissection degree that fell into the best statistical unit in the study area was 0–340.77 m and the raster data were reclassified and divided at 10 m intervals.

**Table 2:** Reclassification of topographic factors.

Topographic Factor	Classification Range of Topographic Factors
Elevation (m)	500–900, 900–1200, 1200–2800, 2800–3200, ≥3200
Slope gradient (°)	0–7, 7–11, 11–30, 30–38, ≥38
Aspect	North, Northeast, East, Southeast, South, Southwest, West, Northwest
Relief (m)	0–100, 100–180, 180–230, ≥230
Dissection (m)	0–30, 30–100, 100–140, ≥140

**Table 3:** Correlation test of topographic factors and gully parameters.

Topographic Factor	Gully Length	Gully Density	Gully Frequency
Elevation	−0.237	0.278	0.580 **
Slope gradient	−0.699 **	0.060	0.269 *
Aspect	−0.061	−0.427	−0.218
Relief	−0.696 **	0.714 **	0.886 **
Dissection	−0.840 **	0.403 *	0.671 **

Note: * and ** indicate *p* < 0.05 and *p* < 0.01, respectively.

## Data Availability

Not applicable.
